# DNA Polymerase β as a Novel Target for Chemotherapeutic Intervention of Colorectal Cancer

**DOI:** 10.1371/journal.pone.0016691

**Published:** 2011-02-02

**Authors:** Aruna S. Jaiswal, Sanjeev Banerjee, Ritu Aneja, Fazlul H. Sarkar, David A. Ostrov, Satya Narayan

**Affiliations:** 1 Department of Anatomy and Cell Biology, College of Medicine, University of Florida, Gainesville, Florida, United States of America; 2 Barbara Ann Karmanos Cancer Institute, Department of Pathology, School of Medicine, Wayne State University, Detroit, Michigan, United States of America; 3 Department of Biology, Georgia State University, Atlanta, Georgia, United States of America; 4 Department of Pathology, Immunology and Laboratory of Medicine, University of Florida, Gainesville, Florida, United States of America; Chinese University of Hong Kong, Hong Kong

## Abstract

Chemoprevention presents a major strategy for the medical management of colorectal cancer. Most drugs used for colorectal cancer therapy induce DNA-alkylation damage, which is primarily repaired by the base excision repair (BER) pathway. Thus, blockade of BER pathway is an attractive option to inhibit the spread of colorectal cancer. Using an *in silico* approach, we performed a structure-based screen by docking small-molecules onto DNA polymerase β (Pol-β) and identified a potent anti-Pol-β compound, NSC-124854. Our goal was to examine whether NSC-124854 could enhance the therapeutic efficacy of DNA-alkylating agent, Temozolomide (TMZ), by blocking BER. First, we determined the specificity of NSC-124854 for Pol-β by examining *in vitro* activities of APE1, Fen1, DNA ligase I, and Pol-β-directed single nucleotide (SN)- and long-patch (LP)-BER. Second, we investigated the effect of NSC-124854 on the efficacy of TMZ to inhibit the growth of mismatch repair (MMR)-deficient and MMR-proficient colon cancer cell lines using *in vitro* clonogenic assays. Third, we explored the effect of NSC-124854 on TMZ-induced *in vivo* tumor growth inhibition of MMR-deficient and MMR-proficient colonic xenografts implanted in female homozygous SCID mice. Our data showed that NSC-124854 has high specificity to Pol-β and blocked Pol-β-directed SN- and LP-BER activities in *in vitro* reconstituted system. Furthermore, NSC-124854 effectively induced the sensitivity of TMZ to MMR-deficient and MMR-proficient colon cancer cells both *in vitro* cell culture and *in vivo* xenograft models. Our findings suggest a potential novel strategy for the development of highly specific structure-based inhibitor for the prevention of colonic tumor progression.

## Introduction

Colorectal cancer is the third most common cancer and the second most common cause of cancer related deaths worldwide [1]. In the year 2010, an estimated 102,900 new colorectal cases will be diagnosed and 51,370 deaths will occur in the United States only. Although in the last two decades an appreciable advancement has been made in the treatment options, the rate of mortality from this disease is not much improved. Therefore, new therapies are needed to improve the prognosis of this disease. For many years, the first choice of chemotherapeutic drug for colorectal cancer has been 5-Fluorouracil (5-FU). It is mostly used as neoadjuvant therapy with radiation and in combination with several other chemotherapeutic drugs, such as mitomycin, cisplatin, oxaliplatin, camptosar, eloxatin, avastin, erbitux, and vectibix for the treatment of colorectal cancer that becomes metastasized [2]. These drugs give best results at higher doses, but cause serious side effects, including the killing of healthy cells of lining of mouth, the lining of the gastrointestinal tract, the hair follicles, the bone marrow and cause liver injury and hypertension [3].

Mutations in the *adenomatous polyposis coli (APC)* gene is an early event in familial adenomatous polyposis (FAP), a syndrome in which there is an inherited predisposition to colon cancer [4], [5]. Most mutations of the *APC* gene occur in the mutation cluster region (MCR) and result in the production of a truncated protein. This truncation compromises several functions of APC, which is involved in chromosomal instability and abnormal functioning of Wnt-signaling pathway, cell cycle regulation, stabilization of the microtubular cytoskeleton, cell-cell interactions and DNA rep y [6]–[14]. Recent studies suggest that the nuclear APC, through a region (amino acids 1441-2077) that is truncated in the majority of colorectal tumors, cooperates in the recruitment of DNAPKcs to the damaged DNA chromatin and enhances early response to double-strand breaks (DSB) DNA repair [15]. APC also interacts directly with genomic DNA, preferentially with A/T rich sequences [16], implying a role for APC in DNA replication [17]. It has been suggested that APC through its C-terminus end (amino acids 2140-2421) interacts with DNA and negatively regulates cell cycle progression through inhibition of DNA replication by direct interaction with DNA [17]. We have previously shown that treatment with DNA-alkylating agents enhances the level of APC in colorectal cancer cells [14]. In addition, we demonstrated that APC interacts with DNA polymerase β (Pol-β) and flap-endonuclease 1 (Fen1) and blocks Pol-β-directed single-nucleotide (SN)- and long-patch (LP)-base excision repair (BER) activities to affect cellular responsiveness to chemotherapy [14], [18]. Based upon these studies, it appears that the interaction of APC with Pol-β and other BER proteins can be an appropriate target for chemotherapeutic intervention of colorectal cancer growth.

The use of DNA-alkylating agents as chemotherapeutic drugs is based on their ability to trigger a cell death response [19], and their therapeutic efficacy is determined by the balance between DNA damage and repair. The DNA-alkylation damage-induced lesions are repaired by the BER, O^6^-methylguanine DNA-methyltransferase (MGMT) and mismatch repair (MMR) pathways. Many colon tumors become resistant to DNA-alkylating agents due to overexpression of MGMT or MMR-deficiency [20]. The cells deficient in MGMT are unable to process the O^6^MeG during DNA synthesis, and if it is not repaired, a G:C to G:T transition mutation occurs [21]. In previous studies, the role of BER pathway has also been implicated in cellular resistance to TMZ [22], [23], which depends on specific BER gene expression and activity [24]. In the past years, the anticancer drugs that have been developed mainly target the MGMT and MMR pathways [25], [26]. Since MMR-deficient colorectal cancers pose a greater risk of resistance to DNA-alkylating drugs due to overexpression of MGMT or MMR-deficiency [27]–[29], it is critical to discover a chemotherapeutic strategy that can be useful for the treatment of both MMR-deficient and MMR-proficient colorectal tumors. Interestingly, although BER is responsible for the repair of 70%, 5% and 9% of N^7^-methylguanine (MeG), N^3^-MeG, and N^3^-methyladenine (MeA) lesions, respectively, induced by the DNA-alkylating drug Temozolomide (TMZ; NSC-362856) [23], [30], [31], the potential utility of BER pathway blockade has not been thoroughly explored. Thus, with the combination of BER blocking agents and TMZ treatment, the clinical outcome of chemotherapeutic efficacy of TMZ can be enhanced. Previous studies also support that DNA alkylation-induced damage is primarily repaired by the BER pathway [26], [27]. In the past, BER pathway has been utilized as a target for the development of new drugs, but the clinical implication of these drugs is still under investigation [31]–[36].

TMZ has been successfully being used for the treatment of metastatic melanoma and glioblastoma multiforme, the latter in combination with radiotherapy [37], [38], but has been shown to be less effective in the treatment of other malignancies. A Phase II clinical study of TMZ in pre-selected advanced aerodigestive tract cancers, including colorectal neoplasm, has been recently completed by Schering-Plough, Kenilworth, NJ, showing only a partial response to the treatment (http://clinicaltrials.gov/ct2/show/NCT00423150). In an earlier Phase I clinical study of TMZ, a partial response of the drug on metastatic colorectal cancer was also observed, suggesting considerable tumor resistance to treatment [39]. To overcome the resistance of TMZ, a Phase II clinical study was performed in which lomeguatrib was combined with TMZ, but the results were not very significant [40]. Thus, there is an urgent need of the development of a new strategy by which the efficacy of TMZ can be increased for the treatment of colorectal cancers.

Since most chemotherapeutic drugs cause DNA damage and resistance due to activation of the DNA repair pathway(s), targeting proteins of these pathway(s) to block their activity can be a promising strategy for the development of novel drugs with higher efficacy to both chemo-resistant and sensitive tumors. In recent years, structure-based virtual design and the three-dimensional structure of a drug-target interaction with small molecular weight inhibitors has been used to guide rationale drug discovery [41]. The computer-aided drug design could find new lead compounds and aided in the structure optimization for biological and pharmacological tests [42]–[44]. In the present study, we performed structure-based molecular docking of Pol-β at the site where adenomatous polyposis coli (APC) interacts and blocks Pol-β-directed BER [14], [45], [46]. The structure-based molecular docking is considered a suitable approach for the development of new drugs, since this approach is targeted to specific protein and specific pathway [47]. Based upon in silico efforts, we have identified a highly potent small molecular weight inhibitor, NSC-124854 {[5-(4-amino-6-iodo-2-oxo-5,6-dihydropyrimidin-1-yl)-3-hydroxy-oxolan-2-yl] methoxyphosphonic acid}, which specifically interacts with Pol-β and blocks Pol-β-directed single-nucleotide (SN)- and long-patch (LP)-BER. Here we present data describing that NSC-124854 can enhance the efficacy of TMZ in MMR-deficient and proficient colon cancer cells *in vitro* and *in vivo* models. We propose that these pre-clinical findings will establish a new paradigm for clinical management of colon cancer.

## Results

### Screening of small molecules to inhibit Pol-β-directed strand-displacement activity

**Figure 1:**
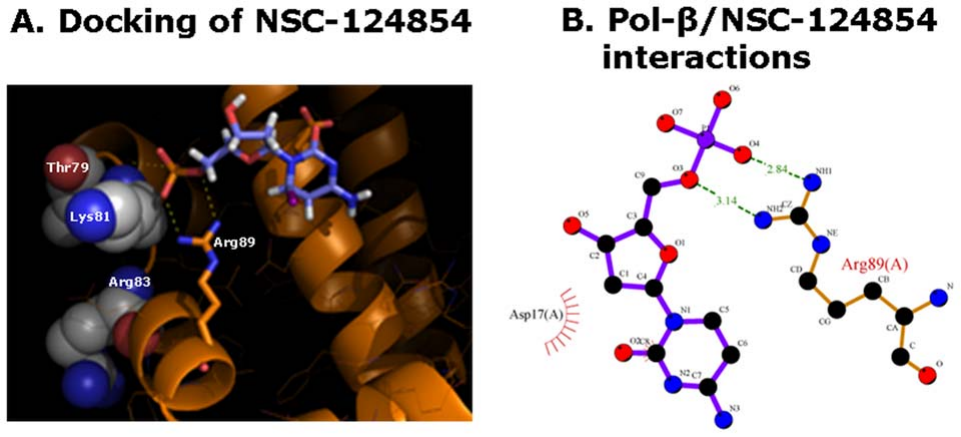
NSC-124854 complex with the simulated structure of Pol-β. **Panel** ***A*** shows the predicted interaction of NSC-124854 based on the crystal structure of Pol-β. Pol-β is shown in gold and side-chains predicted to form contacts with NSC-124854, Asp17 and Arg89, are depicted with gold for carbon, blue for nitrogen and red for oxygen. The APC binding pocket residues, Thr79, Lys81, Arg83 of Pol-β are shown as spheres colored gray for carbon, blue for nitrogen and red for oxygen. Polar contacts are depicted as yellow dashed lines between NSC-124854 and Pol-β residues Lys81 and Arg89. The figure was made with PyMol. **Panel** ***B*** depicts Pol-β-ligand interactions predicted based on the posed molecular docking orientation of NSC-124854. Interactions mediated by hydrogen bonds (green) and by hydrophobic (gray) contacts are shown. NSC-124854 and Pol-β are shown in black for carbon, blue for nitrogen and red for oxygen. Inter-atomic bonds of NSC-124854 are shown in magenta. Inter-atomic bonds of Pol-β are shown in gold. Hydrogen bonds are indicated by dashed lines between the atoms involved, while hydrophobic contacts are represented by an arc with spokes radiating towards the NSC-124854 atoms they contact. The contacted Pol-β atoms are shown with spokes radiating back. The figure was made with HBPLUS and LigPlot.

**Figure 2:**
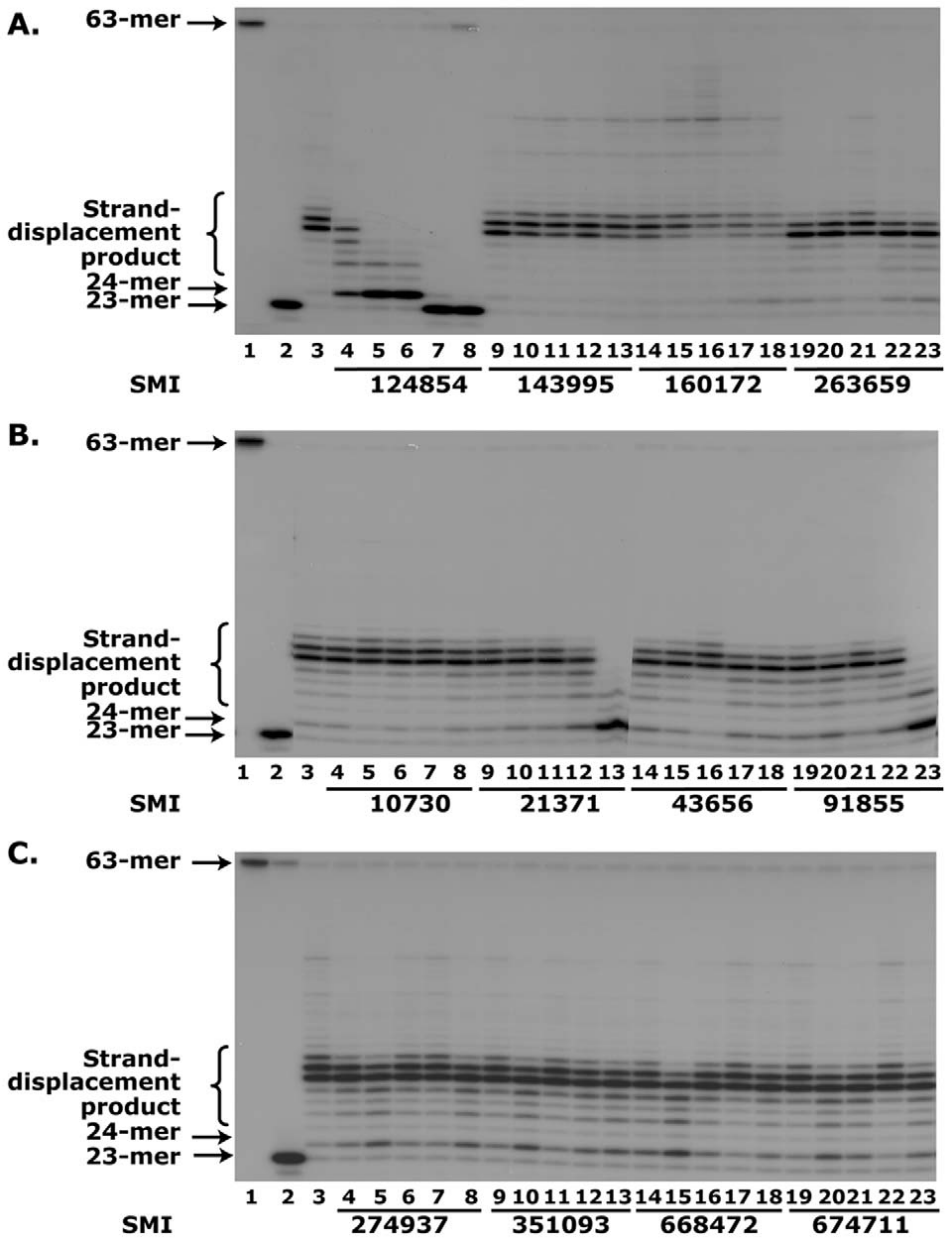
Screening of small molecules. To determine the effect of compounds on the blockage of Pol-β-activity, we assembled *in vitro* strand-displacement synthesis assay with purified APE1 precut ^32^P-labeled 63-mer F-DNA and Pol-β. **Panel** ***A*** (NSC-124854, NSC-143995, NSC-160172 and NSC-263659), ***B*** (NSC-10730, NSC-21371, NSC-43656 and NSC-91855) and ***C*** (NSC-274937, NSC-351093, NSC-668472 and NSC-674711) show the effect of small molecules on Pol-β-directed strand displacement synthesis. In each Panel, lane 1 shows 63-mer ^32^P-labeled F-DNA, lane 2 shows 23-mer product after APE1 incision, lane 3 shows 1-nt incorporation (24-mer) and strand-displacement products. Lanes 4–8, 9–13, 14–18 and 19–23 show the strand-displacement activity of Pol-β incubated with 0, 5, 10, 25, 50 and 125 µM, respectively, of the indicated compounds. Data are representative of two experiments.

To identify a potent anti-Pol-β compound, we used an *in silico* high-throughput structure-based molecular docking approach and screened approximately 140,000 small molecule compounds (molecular weight <500 daltons) for their ability to interact with the APC-binding site of Pol-β (Fig. 1*A*) [48]. The 22 highest scoring small molecular compounds were requested from the Developmental Therapeutics Program (DTP) of National Cancer Institute (NCI) for functional evaluation. We performed the initial screening of the compounds for determining their ability to inhibit Pol-β-directed strand-displacement synthesis. We used an *in vitro* reconsitiuted assay system in these studies. We screened 22 top scoring compounds and the data of 12 compounds is shown here (Fig. 2*A*–*C*). Among 22 small molecules, NSC-21371 and NSC-91855 inhibited Pol-β-directed strand-displacement synthesis at 125 µM concentration (Fig. 2*B*; compare lane 3 with lanes 9–13 and 19–23, respectively). These small molecular compounds did not affect 1-nt incorporation activity (24-mer product) of Pol-β at any tested concentration. However, NSC-124854 inhibited Pol-β-directed strand-displacement activity at 5 µM concentration, while higher concentrations of NSC-124854 completely abrogated the formation of strand-displacement products (Fig. 2*A*; comparison of lane 3 with lanes 4–8 respectively). When the concentration of NSC-124854 was further increased to 50 µM or more, the 1-nt incorporation (24-mer product) activity of Pol-β was completely blocked as shown by the accumulation of 23-mer incision product and lack of 1-nt incorporation product, reflecting a complete loss of Pol-β activity (Fig. 2*A*, compare lane 3 with 4–8, respectively). These results suggest that NSC-124854 is a most potent inhibitor of Pol-β activity among all the compounds tested.

### Small molecule inhibitor, NSC-124854, specifically blocks Pol-β activity

**Figure 3:**
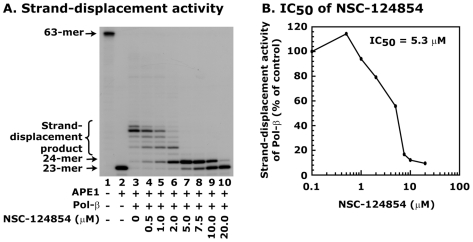
NSC-124854 blocks Pol-β-directed strand-displacement synthesis with a very low IC_50_. To determine the affinity of NSC-124854 for the blockage of Pol-β-directed strand-displacement activity, we determined its IC_50_ in a reconstituted assay system as in described in Figure 2. **Panel** ***A*** shows the autoradiogram of the strand-displacement activity. Lane 1 shows ^32^P-labeled 63-mer F-DNA, lane 2 shows 23-mer product after APE1 incision, and lane 3 shows strand-displacement activity of Pol-β. Lanes 4–10 shows effect of 0.5–20 µM of NSC-124854 on Pol-β activity. **Panel** ***B*** shows the IC_50_ data. NSC-124854 inhibited Pol-β-directed strand-displacement activity in a dose dependent manner with an IC_50_ of 5.3 µM. Data are the representative of two independent estimations.

We further determined whether NSC-124854 is a specific inhibitor of Pol-β activity or it might also block the activity of other BER enzymes. We first determined the IC_50_ of NSC-124854 for Pol-β-directed strand-displacement synthesis. This experiment was the same as described in the screening experiment, except we chose lower concentrations to determine the IC_50_ of NSC-124854. We quantitated the strand-displacement synthesis products (Fig. 3*A*, lanes 3-10, respectively) and plotted as a percent of change of NSC-124854-mediated blockage of Pol-β activity (Fig. 3*B*). The treatment with NSC-124854 showed a dose-dependent decrease in the strand-displacement synthesis activity with an IC_50_ of 5.3 µM (Fig. 3*B*). These results suggest that NSC-124854 has a strong inhibitory effect on Pol-β-directed strand-displacement synthesis.

**Figure 4:**
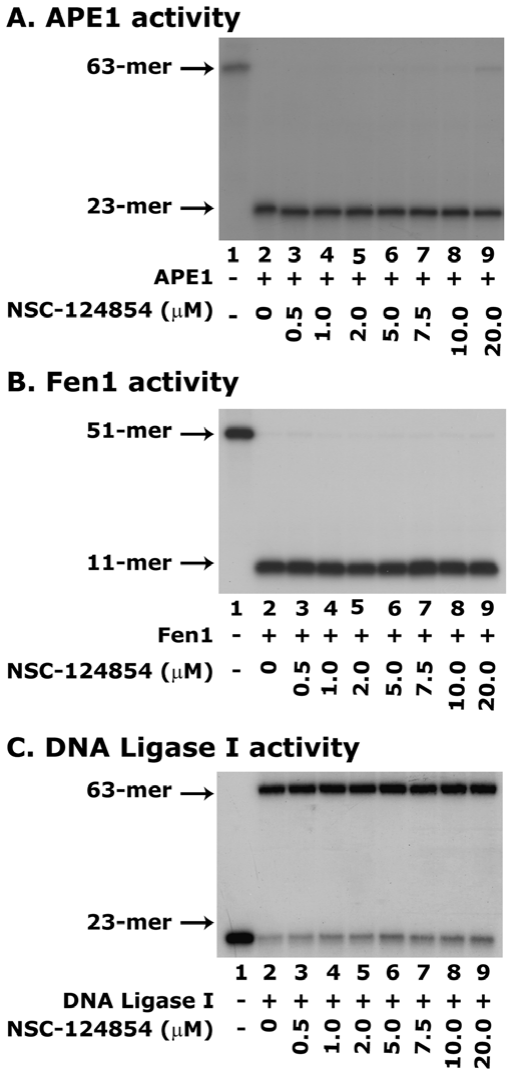
NSC-124854 does not block the activity of APE1, Fen1 and DNA ligase I in reconstituted *in vitro* assays. **Panel** ***A*** shows the effect of NSC-124854 on APE1 activity. APE1 (10 nM) was incubated with different concentrations of NSC-124854 (0.5-20 µM, lanes 3-9, respectively) and ^32^P-labeled 63-mer F-DNA. Lanes 1 and 2 show uncut ^32^P-labeled 63-mer F-DNA and 23-mer incision product, respectively. **Panel** ***B*** shows the effect of NSC-124854 on Fen1 activity. Fen1 (10 nM) was incubated with different concentrations of NSC-124854 (0.5–20 µM, lanes 3–9, respectively) and ^32^P-labeled 51-mer flapped-DNA. Lane 1 shows the position of 51-mer labeled oligonucleotide and lane 2 shows 11-mer cleaved flap product. **Panel** ***C*** shows the effect of NSC-124854 on DNA ligase I activity. DNA ligase I (5 nM) was incubated with different concentrations of NSC-124854 (0.5–20 µM, lanes 3–9, respectively) followed by addition of 2.5 nM of ^32^P-labeled 63-mer nicked DNA. Lane 1 shows 23-mer labeled oligonucleotide (nicked product) and lane 2 shows 63-mer ligated product. Data are representative of two independent experiments.

Next, we determined whether NSC-124854 can inhibit the activity of other BER pathway enzymes such as apurinic/apymidinic endonuclease 1 (APE1), Fen1 and DNA ligase I. Results showed that NSC-124854 did not inihibit the activities of these enzymes (Fig. 4*A*–*C*, respectively). From these results, we concluded that the inhibitory effect of NSC-124854 was highly specific for Pol-β and did not affect the activity of other BER enzymes.

### Small molecule inhibitor NSC-124854 blocks single-nucleotide (SN)- and long-patch (LP)-BER activities in a reconstituted *in vitro* assay

Based upon molecular docking analysis, NSC-124854 is predicted to form polar (H-bonds) interactions with the amino acid residues Lys81 and Arg89 and a non-polar (van der Waals) interaction with the amino acid residue Asp17 on the surface of Pol-β [48], [49]. These residues are in close proximity of the APC binding pocket (amino acid residues Thr79, Lys81 and Arg83) (Fig. 1*B*). These predicted contacts suggest a multiple direct contacts between NSC-124854 and Pol-β, which can possibly mimic the interaction of APC with Pol-β. Since these data suggest that NSC-124854 in combination with TMZ as a possible chemotherapeutic treatment strategy for colorectal cancer, we undertook a study to determine the efficacy and limitations of this strategy.

**Figure 5:**
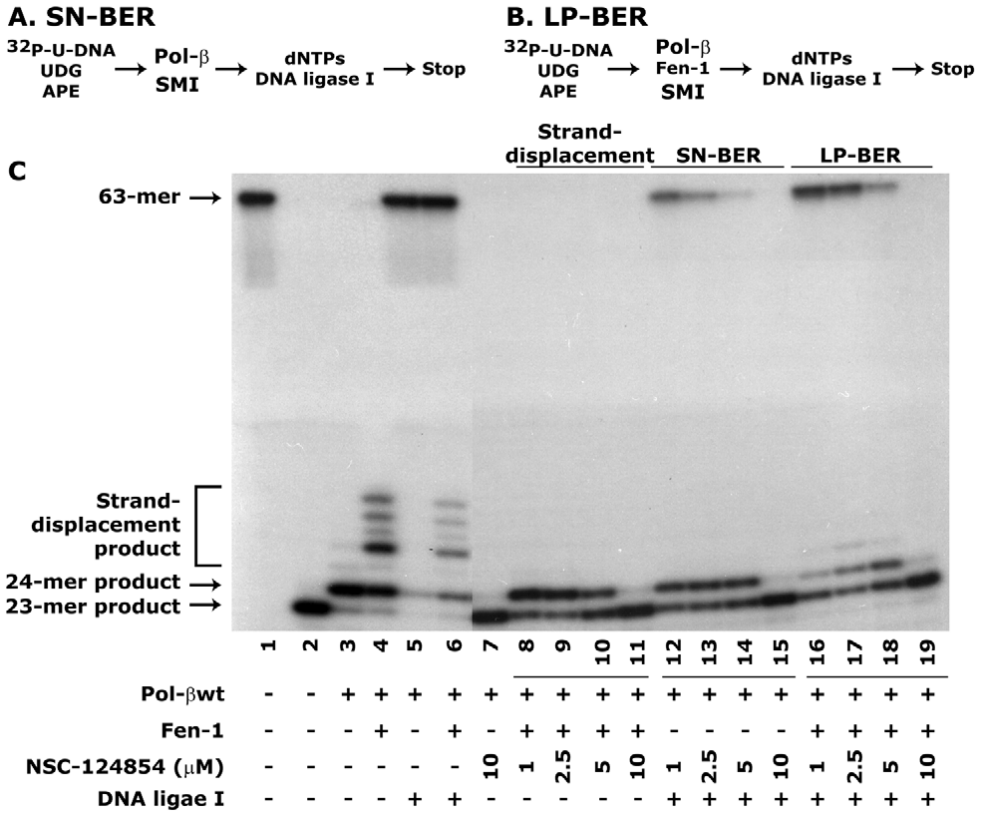
NSC-124854 blocks Pol-β-directed SN- and LP-BER activities. **Panels** ***A and B*** represent the protocols of the SN- and LP-BER, respectively. **Panel** ***C*** shows the autoradiogram describing the effect of varying concentrations of NSC-124854 on SN- and LP-BER activities. Lane 1 shows 63-mer ^32^P-labeled U-DNA, Lane 2 shows 23-mer product after APE1 incision, Lane 3 shows 1-nt incorporation by Pol-β, Lane 4 shows strand-displacement products after the addition of Fen1. Lanes 5 and 6 show the 63-mer ligated product of SN- and LP-BER activities, respectively. Lane 8-19 shows the effect of NSC-124854 on the blockage of 1-nt (24-mer product) incorporation. Lanes 8-11 depict NSC-124854-mediated block of strand-displacement synthesis. Furthermore, lanes 12–15 and lanes 16–19 show the effect of NSC-124854 on the blockage of SN- and LP-BER activities in a dose-dependent manner. Data is a representative of three different experiments.

Although we determined the specificity of NSC-124854 for Pol-β activity as shown in Fig. 3 and 4, it was necessary to examine the effect of this compound when the complete BER system of SN- and LP-BER is assembled. These experiments will provide biochemical evidence for the potency of NSC-124854. We used 63-mer ^32^P-labeled U-DNA as a substrate (Fig. 5*A*) for SN-BER. Since Fen1 is required for LP-BER activity and can stimulate Pol-β activity for strand-displacement synthesis [50], [51], we adopted same strategy for determining the effect of NSC-124854 on LP-BER (Fig. 5*B*). After APE1 incision, an expected 23-mer product was generated (Fig. 5*C*; compare lane 1 with 2). The results showed efficient 1-nt incorporation (24-mer product) by Pol-β which was ligated with DNA ligase I to generate 63-mer repaired product (Fig. 5*C*, lane 5). The addition of Fen1 stimulated Pol-β activity for strand-displacement synthesis (Fig. 5*C*, lane 4), which was also ligated with DNA ligase I to generate 63-mer repaired product (Fig. 5*C*, lane 6). The addition of NSC-124854 blocked Pol-β-directed 1-nt addition (24-mer product in the absence of Fen1) (Fig. 5*C*, compare lane 3 with 7) as well as strand-displacement synthesis in the presence of Fen1 in a dose-dependent manner (Fig. 5*C*, compare lane 4 with lanes 8–11, respectively). Further, the complete repair by SN- and LP-BER sub-pathways after the addition of DNA ligase I was also blocked by NSC-124854 in a dose-dependent manner (Fig. 5*C*, compare lanes 12–15 and 16–19, respectively).

### Expression of wild-type APC causes resistance to TMZ treatment which is abolished by the treatment with NSC-124854

In previous studies, we have shown that APC interacts with Pol-β and Fen1 and blocks SN- and LP-BER activities [14], [45], [46], [52]–[54]. We have also shown that HCT-116 cells (express wild-type APC) are more sensitive to methylmethane sulfonate (MMS) and TMZ treatments than HCT-116-APC(KD) cells (knocked-down APC with pSiRNA) [36], [45], [46]. In the present study, we determined the sensitivity of TMZ in the presence of NSC-124854 to several colon cancer cell lines (HCT-116, HCT-116-APC(KD), HCT-116+ch3, Caco-2, HT29, SW480, LoVo and RKO) using a clonogenic assay [18], [36], [46]. The IC_50_ results showed that all the cell lines tested showed higher sensitivity to the combination treatment of NSC-124854 with TMZ (Table 1). The IC_50_ data showed that NSC-124854 was able to enhance the growth inhibitory effect of TMZ to both HCT-116 and HCT-116-APC(KD) cell lines; however, the effect was greater in HCT-116 than in HCT-116-APC(KD) cells (Table 1). These results confirm our previous findings that wild-type APC by inhibiting BER pathway increases the sensitivity of DNA-alkylaying drugs [36], [46], [54]. However, other cell lines which express either wild-type or truncated APC, such as RKO (310 kDa), SW480 (147 kDa), Caco-2 (150 kDa), LoVo (120 kDa) and HT29 (110 and 200 kDa) did not show a direct correlation of the role of APC in the sensitivity of these drugs. These results suggest that genetic factors other than mutations in the *APC* gene might also play a role in determining the sensitivity of NSC-124854 and TMZ to different colon cancer cells. One of the critical parameters for this differential sensitivity is controlled by the differential status of MMR activity, which is discussed below.

**Table 1:** Effect of NSC-124854 on TMZ-mediated cell growth inhibition of MMR-proficient and MMR-deficient colon cancer cell lines in culture.

	IC_50_ of TMZ (µM)			
NSC-666715 (µM)	0	25	50	100
**MMR-proficient**				
HCT-116+ch3	130.7±4.3	119.7±3.0	88.7±6.4**	68.0±3.6***
Caco-2	449.2±42.4	--	254.1±6.7*	--
HT29	962.7±62.7	--	407.6±23.1***	--
SW480	108.1±3.2	--	43.5±9.6**	--
**MMR-deficient**				
HCT-116	739.0±25.9	644.0±19.3*	468.7±25.8**	344.3±17.2***
HCT-116-APC(KD)	877.7±49.2	744.7±5.3	549.7±2.7**	410.3±6.6***
LoVo	838.8±53.1	--	431.6±103.0*	--
RKO	1572.6±89.9	--	1068.7±195.3	--

Cells were treated with different concentrations of NSC-124854 either alone or in combination with TMZ for 48 h. The cell growth inhibition was determined by the clonogenic assay. The IC_50_ of NSC-124854 for HCT-116, HCT-116-APC(KD) and HCT-116+ch3 is 210 ± 12.0, 251.7±3.3 and 391.4±68.5, respectively. The IC_50_ of NSC-124854 in Caco-2, HT29, SW480, LoVo and RKO was not determinable up to 250 µM of NSC-124854 concentration used in these experiments. Data presented are the mean ± SE of three different estimations. Statistically significant results are shown as asterisks. The p value of the Student's t-test as compared to TMZ treated group is: *, p<0.05; **, p<0.005; and ***, p<0.001.

### Combination treatment of NSC-124854 enhances growth inhibitory effect of TMZ on MMR-deficient and MMR-proficient colon cancer cells

MMR proteins are involved in the chemotherapeutic response of colon cancer cells, and defect in MMR protein(s) is present in the hereditary nonpolyposis colorectal cancer (HNPCC) [55]. Two of the MMR proteins are most commonly mutated in human cancers, MLH1 and MSH2. MMR-deficient cells are frequently resistant to DNA-alkylating agents [55]. To determine the role of MMR in the combination effect of NSC-124854 with TMZ on the growth inhibition, we used several MMR-proficient and MMR-deficient colon cancer cell lines. HCT-116, HCT-116-APC(KD), and RKO cell lines are deficient in MMR due to lack of expression of hMLH1, a key enzyme of this pathway [56], while LoVo cells are MMR-deficient due to absence of MSH2 expression [57]. The HCT-116+ch3 cell has been made MMR-proficient by introducing a single copy of chromosome 3 harboring the *hMLH1* gene [58]. TMZ is known to cause resistance to MMR-deficient cells [26]. In previous studies, it has been suggested that the disruption of BER pathway can abolish drug resistance caused by MMR-deficiency [59]. In the present study, we determined whether treatment of TMZ sensitizes MMR-proficient cells comparatively more than the MMR-deficient cells, and whether the blockade of BER pathway by NSC-124854 can abolish TMZ resistance to MMR-deficient cells. As expected, the MMR-proficient cell lines SW480 (IC_50_ 108.1±3.2 µM), HCT-116+ch3 (IC_50_ 130.7±4.3 µM) and Caco-2 (IC_50_ 449.2±42.4 µM), except HT29 (IC_50_ 962.7±62.7 µM) showed a higher sensitivity to TMZ as compared to MMR-deficient cell lines HCT-116 (IC_50_ 739.0±25.9 µM), HCT-116-APC(KD), (IC_50_ 877.7±49.2 µM), LoVo (IC_50_ 838.8±53.1 µM) and RKO (IC_50_ 1572.6±89.9 µM) (Table 1). Furthermore, the combination treatment of NSC-124854 further reduced the IC_50_ of TMZ by two-fold in all the seven cell lines regardless of their status of MMR activity (Table 1). These results suggest that the combination treatment of NSC-124854 with TMZ could be a useful chemotherapeutic strategy for the management of both MMR-deficient and MMR-proficient colorectal tumors.

### Combination of NSC-124854 with TMZ can be used as a potential chemotherapeutic approach to augment colonic tumor growth *in vivo*

**Figure 6:**
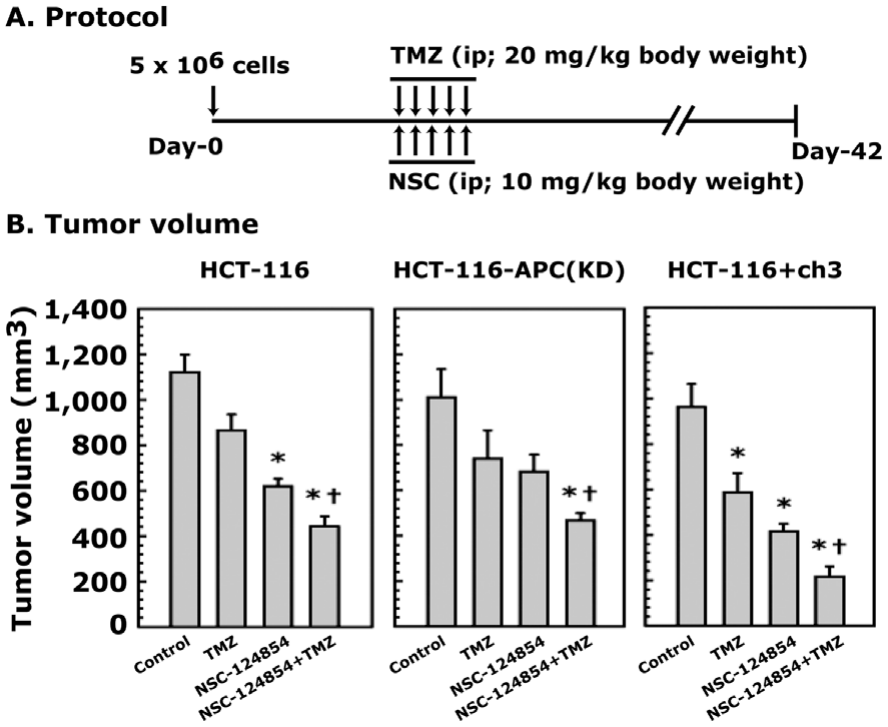
NSC-124854 in combination with TMZ decreases the growth of tumors in a xenograft model. **Panel** ***A*** shows the schematic representation of the experimental protocol. **Panel** ***B*** shows the change in tumor volume at the 42^nd^ day of the experiment. Data presented are the mean ± SD of four to six animals in each group. *, significantly different than control; ^†^, significantly different than NSC-124854. P<0.05.

To further verify the *in vitro* results of the efficacy of the combination treatment of NSC-124854 and TMZ with a well-characterized and specific target of Pol-β to reduce the doses of TMZ that can eliminate side-effects and simultaneously abolish the MMR-resistance, we performed an *in vivo* xenograft study using severe combined immunodeficient (SCID, lacking functional T and B cells) mouse, as described in Figure 6*A*. Our choice for female mice was based on recent study describing that the estimated new cases of colon cancer in 2009 was predicted to be higher in females compared to males [60]. We chose a dose of 20 mg/kg body weight of TMZ for these experiments, which is 10- and 4-times lower than the maximum tolerated dose in mice and human, respectively [61]–[65]. Also, the dose of 10 mg/kg body weight for NSC-124854 is more than 20-times lower than its IC_50_ in culture (Table 1). Although we have not determined the maximum tolerated dose (MTD) of NSC-124854 in mice, the chosen concentration is very much in the safer range.

These drugs were given intraperitoneally (*i.p.*) for five consecutive days. The growth inhibition of tumors was monitored up to 42 days. The results showed an increase in the tumor volume in the control group in a time-dependent manner for all the cell lines, *i.e.*, HCT-116, HCT-116-APC(KD) and HCT-116+ch3. The tumor volume reached a maximum of 1,120 mm^3^ within 42 days of xenograft implantation, which is shown in Figure 6*B*. The tumor growth in the untreated HCT-116 xenograft was higher than HCT-116-APC(KD) and HCT-116+ch3 cells. The anti-tumor effect of TMZ alone was significantly different in HCT-116+ch3 cells (MMR-proficient) and was less pronounced in HCT-116 and HCT-116-APC(KD) cells (Fig. 6*B*). Treatment with NSC-124854 alone also decreased the growth of tumors with all the cell lines, which was more pronounced when it was combined with TMZ (Fig. 6*B*). These results suggest that the combination treatment of NSC-124854 enhances the therapeutic efficacy of TMZ equally well in both MMR-deficient and MMR-proficient xenograft tumor model *in vivo*. To determine the tolerance of the drugs, we recorded the body weight of the animals twice a week until the end of the experiment. The results showed a similar gain in body weight of control and treated group of animals with NSC-124854 and TMZ alone or in combination (data not shown). Thus, it appears that the doses of 10 mg/kg body weight for NSC-124854 and 20 mg/kg body weight for TMZ were well tolerated and did not cause any obvious adverse side effects in our experimental animals.

## Discussion

In previous studies, Pol-β has been used as a target for chemotherapeutic drug development [23], [34], but did not reach beyond the pre-clinical level. The efficacy of the previous compounds has been less effective because they require very high concentrations to achieve the desired cytotoxicity *in vitro* and they were not tested systematically *in vivo*. Furthermore, they were mainly targeted to block SN-BER. The use of TMZ for the treatment of malignancies other than glioblastoma and melanoma has been limited, especially for the treatment of colorectal tumors, due to less pronounced effect on suppression of tumor growth [39], [40]. In the present study, we used structure-based molecular approach to identify small molecule which can mimic the interaction of APC with Pol-β and block Pol-β-directed BER that can be utilized as a chemotherapeutic target. Our strategy of molecular docking in the APC-binding pocket of Pol-β was to identify a small molecule which can block both SN- and LP-BER activities and demonstrate cytotoxicity in both *in vitro* and *in vivo* assays at lower concentrations.

Notably, if successful, the proposed strategy will be highly effective in the prevention of both MMR-proficient and MMR-deficient colorectal cancers; this is of importance because the MMR-deficient colorectal cancers pose a greater risk of resistance to DNA-alkylating drugs due to over-expression of MGMT or MMR-deficiency [27]–[29]. The cells deficient in MGMT are unable to process the O^6^MeG during DNA synthesis, and if unrepaired, a G:C to G:T transition mutation occurs [27]. The G:T mismatch is then repaired by MMR pathway [28]. However, if the O^6^MeG is not repaired before the re-synthesis step in MMR, the thymine is likely to be re-inserted opposite to the lesion. It is believed that the repetitive cycle of futile MMR results into a generation of tertiary lesions, most likely gapped DNA. This then gives rise to double-stranded breaks (DSBs) in DNA that elicits a cell death response [29], [66]. Furthermore, it has been shown that TMZ causes resistance to MMR-deficient colonic tumors and limits its use in the treatment of these cancers [67]. Thus, a chemotherapeutic strategy that can induce cell death in both MMR-proficient and MMR-deficient colon cancer cells is highly desirable [68]. Our results indicate that the strategy of combining NSC-124854 with TMZ seems to be effectively blocking the growth of both MMR-proficient and MMR-deficient colon cancer cells *in vitro* and causing anti-tumor activity *in vivo,* which supports the previous findings [27], [59]. This suggests that the blockade of the repair of TMZ-induced N^7^-MeG, N^3^-MeG, N^3^-MeA lesions (non-mutagenic) by NSC-124854 causes much higher cytotoxicity than the mutagenic lesions of O^6^-MeG. Thus, our approach of combining NSC-124854 with TMZ shows promise in that NSC-124854 can overcome TMZ-induced resistance at lower doses and increase its efficacy against colorectal cancer. Since our strategy is to block Pol-β activity by small molecular compounds and increase the efficacy of TMZ, it will also benefit patients who carry wild-type or mutant *APC* gene; the mutant APC gene is the precursors of adenomas and carcinomas in familial adenomatous polyosis (FAP) colon cancer.

Based on our *in vitro* and *in vivo* results there are certain points which need further discussion regarding the tumor growth inhibition after drug treatments. In the *in vitro* studies, we clearly demonstrated that APC caused sensitization of HCT-116 cells than HCT-116-APC(KD) cells. Also, the MMR-proficient HCT-116+ch3 cells were more sensitive to TMZ treatment than MMR-deficient HCT-116 and HCT-116-APC(KD) cells. These *in vitro* results did not translate in a similar fashion to *in vivo* results in which the effect of TMZ on MMR-proficient and MMR-deficient xenografts was not as prominent as was seen with *in vitro* assays. It appears that the dose of TMZ (20 mg/kg body weight) used for *in vivo* studies, perhaps, was high enough to show the difference in the sensitivity of MMR-proficient and MMR-deficient cells. A similar observation has been made in previous studies as well where even up to two-fold higher concentration of TMZ (40 mg/kg body weight) did not show significant difference on the xenograft tumor growth inhibition of MMR-proficient and MMR-deficient colon cancer cells [33]. Another point which we noted in these studies was the effect of NSC-124854 alone on the decrease of growth of xenograft tumors, although it was significant only with HCT-116+ch3 tumors. We initially predicted that NSC-124854 being specific for Pol-β will show no effect on tumor growth if treated alone. However, the effect of NSC-124854 alone on the growth of xenograft tumors observed in this study could be due to the production of more abasic lesions than cells in culture, which sensitizes these xenograft tumor cells to the treatment with NSC-124854. Also, it is possible that NSC-124854 blocks critical cell survival pathways which are active in xenograft tumors but not in cultured cells. The doses of NSC-124854 (10 mg/kg body weight) and TMZ (20 mg/kg body weight) which we used in these studies, seems to be well tolerated by the animals. However, it will be necessary in the future to determine the maximum tolerated dose (MTD) of NSC-124854 alone and together with TMZ so that a highest efficacy of these drugs can be translated into clinical practice. Importantly, although these studies are focused on a lead small molecular compound, NSC-124854, it provides insight into the rational development of second generation small molecular compounds with greater potency and specificity for the treatment of colon cancer. The results allow us to discuss the “proof-of-principle” by which Pol-β-targeted compounds enhance the efficacy of TMZ. Thus, by developing a target-defined strategy of chemotherapy and with the appropriate understanding of the mechanism of action, the strategy of combination of NSC-124854 with TMZ may provide a foundation for both clinical and scientific development of colon cancer management.

## Materials and Methods

### Cell lines

Human colon cancer cell lines HCT-116, SW480, RKO, Caco-2, LoVo and HT29 were obtained from ATCC (Manassas, VA). HCT-116-APC(KD) cell line was established in our laboratory [52], and HCT-116+ch3 cell line was a gift from Dr. Tom Kunkel (NIEHS, Research Triangle Park, NC) [69]. HCT-116, HCT-116-APC(KD) and HCT-1116+ch3 cells were grown in McCoy' 5a, RKO and Caco-2 cells were grown in MEM, and LoVo cells were grown in Ham's F-12 medium at 37°C under a humidified atmosphere of 5% CO_2_. For each cell line, the medium was supplemented with 10% fetal bovine serum (Hyclone, Logan, UT) 100 U/ml of penicillin, and 100 µg/ml of streptomycin.

#### Oligonucleotides and Chemicals

All oligonucleotides were purchased from Sigma-Genosys (Woodlands, TX). Restriction enzymes and T4-polynucleotide kinase (PNK) was purchased from New England Biolabs (Ipswich, MA) and radionuclide [γ-^32^P]ATP was purchased from Perkin Elmer, Inc. (Boston, MA).

### Molecular docking

The molecular docking was performed using the atomic coordinates extracted from the crystal structure of human Pol-β (PDB code 1BPZ) as described in our previous studies [36]. Approximately 140,000 small molecules from the NCI/DTP database were positioned in the selected structural pocket (which includes amino acid residues T79/K81/R83 of Pol-β) and scored based on predicted polar (H-bond) and non-polar (van der Waals) interactions. Each of the small molecules was positioned in the selected site in 100 different orientations. The best orientation and scores (contact and electrostatic) were calculated. The grid based scoring algorithm approximates van der Waals and H bond interactions. Ten mg of the 22 highest-scoring compounds for the selected structural pocket were obtained for use in Pol-β inhibition assays from the NCI/DTP.

#### Screening of small molecules to examine blockade of Pol-β-directed displacement activity

To identify a potent anti-Pol-β compound, we screened top 22 scoring small molecular compounds for determining their ability to block Pol-β activity for functional evaluation. We used *in vitro* Pol-β-directed strand-displacement activity assay for the initial screening of the compounds. Briefly, the strand-displacement reaction mixture was assembled in 30 µl volume with 30 mM Hepes, pH 7.5, 30 mM KCl, 8.0 mM MgCl_2_, 1.0 mM DTT, 100 µg/ml BSA, 0.01% (v/v) Nonidet P-40, 2.5 nM of ^32^P-labeled 63-mer F-DNA substrate, 2 nM of APE, 5 nM of Pol-β and 0-125 µM of NSC-124854. The reaction mixture was incubated for 30 min at 37°C and terminated by the addition of 30 µl of stop solution (5.0 mM of EDTA, 0.4% (w/v) SDS). DNA was extracted with an equal volume of phenol/chloroform/isoamyl alcohol (25∶24∶1, v/v) followed by ethanol precipitation. The strand-displacement products were resolved on a 15% polyacrylamide-7 M urea gel.

#### APE1 assay

The *in vitro* APE1 activity was determined in the presence and absence of different concentrations of NSC-124854. The reaction mixture in a 30 µl volume contained 30 mM of Hepes, pH 7.5; 30 mM of KCl, 8.0 mM of MgCl_2_, 1.0 mM of DTT and 100 µg/ml of BSA. Briefly, 5 nM of APE1 was incubated with 0-20 µM of NSC-124854 and reaction was initiated by addition of 2.5 nM of ^32^P-labeled 63-mer F-DNA substrate. Reaction was incubated for 20 min at 37°C and terminated by the addition of 30 µl of stop solution (5.0 mM of EDTA, 0.4% (w/v) SDS). DNA was extracted with an equal volume of phenol/chloroform/isoamyl alcohol (25∶24∶1, v/v) followed by ethanol precipitation. APE1 incised product was resolved on a 15% polyacrylamide-7 M urea gel.

#### Fen1 endonuclease assay

The *in vitro* Fen1 activity was determined in the presence and absence of different concentrations of NSC-124854. The reaction mixture in a 30 µl volume contained 30 mM of Hepes, pH 7.5; 30 mM of KCl, 8.0 mM of MgCl_2_, 1.0 mM of DTT and 100 µg/ml of BSA [18], [52]. Briefly, 10 nM of Fen1 was incubated with 0-20 µM of NSC-124854 at room temp for 5 min followed by addition 2.5 nM of ^32^P-labeled 51-mer flapped-DNA substrate. Reaction was allowed to proceed for 20 min at 37°C and terminated by the addition of 20 µl of stop solution (5.0 mM of EDTA, 0.4% (w/v) SDS). DNA was extracted with an equal volume of phenol/chloroform/isoamyl alcohol (25∶24∶1, v/v) followed by ethanol precipitation. The 11-mer reaction products were resolved on a 15% polyacrylamide-7 M urea gel.

#### DNA ligase I assay

The *in vitro* DNA ligase I activity was determined in the presence and absence of different concentrations of NSC-124854. The reaction mixture in a 30 µl volume contained 30 mM of Hepes, pH 7.5; 30 mM of KCl, 8.0 mM of MgCl_2_, 1.0 mM of DTT and 100 µg/ml of BSA as described earlier [52]. Briefly, 5 nM of DNA ligase I was incubated with 0–20 µM of NSC-124854 at room temp for 5 min followed by addition 2.5 nM of ^32^P-labeled nicked-DNA substrate. Reaction was incubated for 60 min at 37°C and terminated by the addition of 30 µl of stop solution (5.0 mM of EDTA, 0.4% (w/v) SDS). DNA was extracted with an equal volume of phenol/chloroform/isoamyl alcohol (25∶24∶1, v/v) followed by ethanol precipitation. The reaction products were resolved on a 15% polyacrylamide-7 M urea gel.

### *In vitro* BER assays with purified proteins

The procedure for BER assays were essentially the same as described in our previous studies [18], [36], [45], [46], [52]. Briefly, the BER reaction mixture contained 30 mM Hepes, pH 7.5, 30 mM KCl, 8.0 mM MgCl_2_, 1.0 mM DTT, 100 µg/ml BSA, 0.01% (v/v) Nonidet P-40, 0.5 mM ATP, and 20 µM each of dATP, dCTP, dGTP, dTTP in a final volume of 20 µl. The following additions were made to the above mixture: (*i*) For SN-BER, 2.5 nM of ^32^P-labeled 63-mer U-DNA (containing uracil at the 24^th^ position, pre-incubated with 1 unit of UDG to create an abasic site and 1 nM APE1 to create an incision at the 5′ end of the repair site), 5 nM of Pol-β and different concentrations of NSC-124854; and (*ii*) For LP-BER, the reaction mixture was assembled similarly as for SN-BER, except Fen1 was included in the reaction mixture. The repair was initiated with the addition of 0.4 nM of DNA ligase I.

### Clonogenic assays

A single cell suspension of HCT-116, HCT-116-APC(KD), and HCT-116+ch3 cells were plated 200 cells/well, while HT29, SW480, LoVo, Caco-2 and RKO cells were plated 400 cells/well in triplicate in a six-well plates. Cells were pretreated for 1 h with NSC-124854 or vehicle (0.1% DMSO) followed by treatment with varying concentrations of TMZ for 48 h. After the treatment, the culture medium was replaced with fresh medium and cells were allowed to grow for an additional 8 days. Visible colonies of more than 50 cells were stained with methylene blue and counted for viability [18], [36], [46].

### Xenograft studies

Female homozygous, 6-week old, SCID mice purchased from Taconic Farms, Inc., were used in the study. Prior to initiate the animal experiment, the animal protocol was submitted to the Animal Investigation Committee (AIC) of Wayne State University. This is the Institutional Review Board called AIC Committee who approves animal protocols. All other animal-related research approvals including ethics are also obtained through this committee. The protocol was approved in March 6, 2008 with the protocol number A04-06-08, which will expire on April 30, 2011. HCT-116, HCT-116-APC(KD) and HCT-116+ch3 cells were harvested and a single cell suspension with >95% viability (5x10^6^ cells) diluted in equal volume of Matrigel (BD Biosciences) were injected subcutaneously into the right flank of each mouse. After the tumors were established, as determined by caliper measurements (xenograft tumor volumes were approximately 50–75 mm^3^ after 10 days of cell injection), the mice were randomized into the following six groups (n = 6–4): (*a*) Vehicle control, (*b*) TMZ, (*c*) NSC-124854, and (*d*) NSC-124854 + TMZ. The doses for NSC-124854 and TMZ were 10 mg/kg body weight and 20 mg/kg body weight, respectively. Drugs were administered intraperitoneally (*i.p.*) every day for 5 consecutive days. Tumor volume was measured weekly in each group. All mice were euthanized when the tumor volume in the control mice reached approximately 1,000 mm^3^ (day 42). The mice were housed and maintained under sterile conditions in facilities accredited by the American Association for the Accreditation of Laboratory Animal Care and in accordance with current regulations and standards of the United States Department of Agriculture, United States Department of Health and Human Services, and the NIH.

### Statistical analysis

The statistical significance between experimental groups and control was determined by Student's ‘*t’* test. For mouse xenograft study the statistical significance of differential findings between experimental groups and control was determined by two-way ANOVA as implemented by GraphPad StatMate (GraphPad Software, La Jolla, CA). P<0.05 was considered statistically significant.

## References

[1] Jemal A, Siegel R, Xu J, Ward E (2010). Cancer statistics, 2010.. CA Cancer J Clin.

[2] Abbas A, Yang G, Fakih M (2010). Management of anal cancer in 2010. Part 2: current treatment standards and future directions.. Oncology (Williston Park).

[3] Thompson PA, Gerner EW (2009). Current concepts in colorectal cancer prevention.. Expert Rev Gastroenterol Hepatol.

[4] Miyoshi Y, Nagase H, Ando H, Horii A, Ichii S (1992). Somatic mutations of the *APC* gene in colorectal tumors: mutation cluster region in the *APC* gene.. Hum Mol Genet.

[5] Powell SM, Zilz N, Beazer-Barclay Y, Bryan TM, Hamilton SR (1992). APC mutations occur early during colorectal tumorigenesis.. Nature.

[6] Fodde R, Kuipers J, Rosenberg C, Smits R, Kielman M (2001). Mutations in the APC tumor suppressor gene cause chromosomal instability.. Nat Cell Biol.

[7] Goss KH, Groden J (2000). Biology of the adenomatous polyposis coli tumor suppressor.. J Clin Oncol.

[8] Fearnhead NS, Britton MP, Bodmer WF (2001). The ABC of APC.. Hum Mol Genet.

[9] Narayan S, Roy D (2003). Role of APC and DNA mismatch repair genes in the development of colorectal cancers.. Mol Cancer.

[10] Ishidate T, Matsumine A, Toyoshima K, Akiyama T (2000). The APC-hDLG complex negatively regulates cell cycle progression from the G_o_/G_1_ to S phase.. Oncogene.

[11] Kaplan KB, Burds AA, Swedlow JR, Bekir SS, Sorger PK (2001). A role for the Adenomatous Polyposis Coli protein in chromosome segregation.. Nat Cell Biol.

[12] Green RA, Wollman R, Kaplan KB (2005). APC and EB1 function together in mitosis to regulate spindle dynamics and chromosome alignment.. Mol Biol Cell.

[13] Barth AIM, Pollack AL, Altschuler Y, Mostov KE, Nelson WJ (1997). NH_2_-terminal deletion of β-catenin results in stable colocalization of mutant β-catenin with adenomatpous polyposis coli protein and altered MDCK cell adhesion.. J Cell Biol.

[14] Jaiswal AS, Narayan S (2008). A novel function of adenomatous polyposis coli (APC) in regulating DNA repair.. Cancer Lett.

[15] Kouzmenko AP, Takeyama K, Kawasaki Y, Akiyama T, Kato S (2008). Truncation mutations abolish chromatin-associated activities of adenomatous polyposis coli.. Oncogene.

[16] Deka J, Herter P, Sprenger-Haussels M, Koosch S, Franz D (1999). The APC protein binds to A/T rich DNA sequence.. Oncogene.

[17] Qian J, Sarnaik A, Bonney T, Keirsey J, Combs K (2008). The APC tumor suppressor inhibits DNA replication by directly binding to DNA via its carboxyl terminus.. Gastroenterology.

[18] Panda H, Jaiswal AS, Corsino PE, Armas ML, Law BK (2009). Amino acid Asp181 of 5′-flap endonuclease 1 is a useful target for chemotherapeutic development.. Biochemistry.

[19] Sawyers C (2004). Targeted Cancer Therapy.. Nature.

[20] Liu L, Gerson SL (2006). Targeted modulation of MGMT: clinical implications.. Clin Cancer Res.

[21] Kaina B, Ochs K, Grosch S, Fritz G, Lips J (2001). BER, MGMT, and MMR in defense against alkylation-induced genotoxicity and apoptosis. Prog.. Nucleic Acid Res Mol Biol.

[22] Liu L, Taverna P, hitacre CM, Chatterjee S, Gerson SL (1999). Pharmacologic disruption of base excision repair sensitizes mismatch repair-deficient and -proficient colon cancer cells to methylating agents.. Clin Cancer Res.

[23] Trivedi RN, Almeida KH, Fornsaglio JL, Schamus S, Sobol RW (2005). The role of base excision repair in the sensitivity and resistance to temozolomide-mediated cell death.. Cancer Res.

[24] Trivedi RN, Wang XH, Jelezcova E, Goellner EM, Tang JB (2008). Human methyl purine DNA glycosylase and DNA polymerase beta expression collectively predict sensitivity to temozolomide.. Mol Pharmacol.

[25] Karran P, Bignami M (1994). DNA damage tolerance, mismatch repair and genome instability.. Bioessays.

[26] Liu L, Markowitz S, Gerson SL (1996). Mismatch repair mutations override alkyltransferase in conferring resistance to temozolomide but not to 1,3-bis(2-chloroethyl)nitrosourea.. Cancer Res.

[27] Branch P, Aquilina G, Bignami M, Karran P (1993). Defective mismatch binding and a mutator phenotype in cells tolerant to DNA damage.. Nature.

[28] Kawate H, Sakumi K, Tsuzuki T, Nakatsuru Y, Ishikawa T (1998). Separation of killing and tumorigenic effects of an alkylating agent in mice defective in two of the DNA repair genes.. Proc Natl Acad Sci USA.

[29] Tentori L, Lacal PM, Benincasa E, Franco D, Faraoni I (1998). Role of wild-type p53 on the antineoplastic activity of temozolomide alone or combined with inhibitors of poly(ADP-ribose) polymerase.. J Pharmacol Exp Ther.

[30] Fishel ML, He Y, Smith ML, Kelley MR (2007). Manipulation of base excision repair to sensitize ovarian cancer cells to alkylating agent temozolomide.. Cancer Ther Preclin.

[31] Liu L, Gerson SL (2004). Therapeutic impact of methoxyamine: blocking repair of abasic sites in the base excision repair pathway.. Curr Opin Investig Drugs.

[32] Sobol RW, Wilson SH (2001). Mammalian DNA beta-polymerase in base excision repair of alkylation damage.. Prog Nucleic Acid Res Mol Biol.

[33] Liu L, Nakatsuru Y, Gerson SL (2002). Base excision repair as a therapeutic target in colon cancer.. Clin Cancer Res.

[34] Horton JK, Wilson SH (2007). Hypersensitivity phenotypes associated with genetic and synthetic inhibitor-induced base excision repair deficiency.. DNA Repair (Amst).

[35] Adhikari S, Choudhury S, Mitra PS, Dubash JJ, Sajankila SP (2008). Targeting base excision repair for chemosensitization.. Anticancer Agents Med Chem.

[36] Jaiswal AS, Banerjee S, Panda H, Bulkin CD, Izumi T (2009). A novel inhibitor of DNA polymerase beta enhances the ability of temozolomide to impair the growth of colon cancer cells.. Mol Cancer Res.

[37] Bei R, Marzocchella L, Turriziani M (2010). The use of temozolomide for the treatment of malignant tumors: clinical evidence and molecular mechanisms of action. Recent Pat.. Anticancer Drug Discov.

[38] Hainsworth JD, Ervin T, Friedman E, Priego V, Murphy PB (2010). Concurrent radiotherapy and temozolomide followed by temozolomide and sorafenib in the first-line treatment of patients with glioblastoma multiforme.. Cancer.

[39] Spiro TP, Liu L, Majka S, Haaga J, Willson JK (2001). Temozolomide: the effect of once- and twice-a-day dosing on tumor tissue levels of the DNA repair protein O(6)-alkylguanine-DNA-alkyltransferase.. Clin Cancer Res.

[40] Khan OA, Ranson M, Michael M, Olver I, Levitt NC (2008). Mortimer P, Watson AJ, Margison GP, Midgley R, Middleton MR. A phase II trial of lomeguatrib and temozolomide in metastatic colorectal cancer.. Br J Cancer.

[41] Barcellos GB, Pauli I, Caceres RA, Timmers LF, Dias R (2008). Molecular modeling as a tool for drug discovery.. Curr Drug Targets.

[42] Veselovsky AV, Ivanov AS (2003). Strategy of computer-aided drug design.. Curr Drug Targets Infect Disord.

[43] Kroemer RT (2007). Structure-based drug design: docking and scoring.. Curr Protein Pept Sci.

[44] Dhaliwal B, Chen YW (2009). Computational resources for protein modelling and drug discovery applications.. Infect Disord Drug Targets.

[45] Narayan S, Jaiswal AS, Balusu R (2005). Tumor suppressor APC blocks DNA polymerase beta-dependent strand displacement synthesis during long patch but not short patch base excision repair and increases sensitivity to methylmethane sulfonate.. J Biol Chem.

[46] Balusu R, Jaiswal AS, Armas ML, Bloom LB, Narayan S (2007). Structure/function analysis of the interaction of adenomatous polyposis coli (APC) with DNA polymerase β and its implications for base excision repair.. Biochemistry.

[47] De Azevedo WF (2010). MolDock applied to structure-based virtual screening.. Curr Drug Targets.

[48] Sawaya MR, Prasad R, Wilson SH, Kraut J, Pelletier H (1997). Crystal structures of human DNA polymerase beta complexed with gapped and nicked DNA: evidence for an induced fit mechanism.. Biochemistry.

[49] Wallace AC, Laskowski RA, Thornton JM (1996). LIGPLOT: a program to generate schematic diagrams of protein-ligand interactions.. Protein Eng.

[50] Prasad R, Dianov GL, Bohr VA, Wilson SH (2000). FEN1 stimulation of DNA polymerase beta mediates an excision step in mammalian long patch base excision repair.. J Biol Chem.

[51] Liu Y, Beard WA, Shock DD, Prasad R, Hou EW (2005). DNA polymerase beta and flap endonuclease 1 enzymatic specificities sustain DNA synthesis for long patch base excision repair.. J Biol Chem.

[52] Jaiswal AS, Balusu R, Armas ML, Kundu CN, Narayan S (2006). Mechanism of adenomatous polyposis coli (APC)-mediated blockage of long-patch base excision repair.. Biochemistry.

[53] Kundu CN, Balusu R, Jaiswal AS, Gairola CG, Narayan S (2007). Cigarette smoke condensate-induced levels of adenomatous polyposis coli (APC) block long-patch base excision repair in breast epithelial cells.. Oncogene.

[54] Kundu CN, Balusu R, Jaiswal AS, Narayan S (2007). Adenomatous polyposis coli-mediated hypersensitivity of mouse embryonic fibroblast cell lines to methylmethane sulfonate treatment: implication of base excision repair pathways.. Carcinogenesis.

[55] Taverna P, Liu L, Hanson AJ, Monks A, Gerson SL (2000). Characterization of MLH1 and MSH2 DNA mismatch repair proteins in cell lines of the NCI anticancer drug screen.. Cancer Chemother Pharmacol.

[56] Papadopoulos N, Nicolaides NC, Wei YF, Ruben SM, Carter KC (1994). Mutation of a mutL homolog in hereditary colon cancer.. Science.

[57] Jacob S, Aguado M, Fallik D, Praz F (2001). The role of the DNA mismatch repair system in the cytotoxicity of the topoisomerase inhibitors camptothecin and etoposide to human colorectal cancer cells.. Cancer Res.

[58] Koi M, Umar A, Chauhan DP, Cherian SP, Carethers JM (1994). Human chromosome 3 corrects mismatch repair deficiency and microsatellite instability and reduces N-methyl-N'-nitro-N-nitrosoguanidine tolerance in colon tumor cells with homozygous hMLH1 mutation.. Cancer Res.

[59] Liu L, Taverna P, hitacre CM, Chatterjee S, Gerson SL (1999). Pharmacologic disruption of base excision repair sensitizes mismatch repair-deficient and -proficient colon cancer cells to methylating agents.. Clin Cancer Res.

[60] Jemal A, Siegel R, Ward E, Hao Y, Xu J (2009). Cancer statistics, 2009.. CA Cancer J Clin.

[61] Newlands ES, Blackledge GR, Slack JA, Rustin GJ, Smith DB (1992). Phase I trial of temozolomide (CCRG 81045: M&B 39831: NSC 362856).. Br J Cancer.

[62] Brandes AA, Ermani M, Basso U, Paris MK, Lumachi F (2002). Temozolomide in patients with glioblastoma at second relapse after first line nitrosourea-procarbazine failure: a phase II study.. Oncology.

[63] Seiter K, Liu D, Loughran T, Siddiqui A, Baskind P (2002). Phase I study of temozolomide in relapsed/refractory acute leukemia.. J Clin Oncol.

[64] Trudeau ME, Crump M, Charpentier D, Yelle L, Bordeleau L (2010). Temozolomide in metastatic breast cancer (MBC): a phase II trial of the National Cancer Institute of Canada - Clinical Trials Group (NCIC-CTG).. Ann Oncol.

[65] Cai W, Maldonado NV, Cui W, Harutyunyan N, Ji L (2010). Activity of irinotecan and temozolomide in the presence of O6-methylguanine-DNA methyltransferase inhibition in neuroblastoma pre-clinical models Br J Cancer.

[66] Kaina B, Christmann M, Naumann S, Roos WP (2007). MGMT: key node in the battle against genotoxicity, carcinogenicity and apoptosis induced by alkylating agents.. DNA Repair (Amst).

[67] Taverna P, Liu L, Hwang HS, Hanson AJ, Kinsella TJ (2001). Methoxyamine potentiates DNA single strand breaks and double strand breaks induced by temozolomide in colon cancer cells.. Mutat Res.

[68] Sarkaria JN, Kitange GJ, James CD, Plummer R, Calvert H (2008). Mechanisms of chemoresistance to alkylating agents in malignant glioma.. Clin Cancer Res.

[69] Koi M, Umar A, Chauhan DP, Cherian SP, Carethers JM (1994). Human chromosome 3 corrects mismatch repair deficiency and microsatellite instability and reduces N-methyl-N'-nitro-N-nitrosoguanidine tolerance in colon tumor cells with homozygous hMLH1 mutation.. Cancer Res.

